# Prevalence and diversity of *Eimeria* spp. in free-range chickens in northeastern Brazil

**DOI:** 10.3389/fvets.2022.1031330

**Published:** 2022-10-13

**Authors:** Juliana Trajano da Silva, Felipe Boniedj Ventura Alvares, Estefany Ferreira de Lima, Geraldo Moreira da Silva Filho, Ana Luzia Peixoto da Silva, Brendo Andrade Lima, Thais Ferreira Feitosa, Vinícius Longo Ribeiro Vilela

**Affiliations:** ^1^Programa de Pós-Graduação em Ciência Animal, Universidade Federal de Campina Grande - UFCG, Campina Grande, Paraíba, Brazil; ^2^Departamento de Medicina Veterinária, Instituto Federal da Paraíba - IFPB, Sousa, Paraíba, Brazil

**Keywords:** aviculture, coccidia, eimeriosis, protozoosis, semiarid

## Abstract

In tropical regions, family farming is a form of production and work that is highly present in rural areas. Because the production system for free-range chickens has a low level of technification, it frequently presents massive infection by coccidia. The objective of this study was to determine the prevalence and diversity of *Eimeria* species in free-range chickens in northeastern Brazil. Fecal analyses were carried out using materials collected from 100 farms, belonging to 10 different municipalities. The sample from each farm was composed of five stool samples collected from different animals. Coproparasitological analyses were performed and, in each positive sample, photomicrographs of 20 oocysts were used for morphological identification of coccidia. The presence of *Eimeria* spp. was detected in 59% (59/100) of the farms analyzed. Species identification was performed through morphometric analysis of 1,180 sporulated oocysts. The following eight species of *Eimeria* spp. were found, in decreasing order of prevalence: *Eimeria necatrix* (25%), *Eimeria mitis* (18.3%), *Eimeria mivati* (17.3%), *Eimeria tenella* (12.4%), *Eimeria brunetti* (9.9%), *Eimeria acervulina* (9.1%), *Eimeria praecox* (4.8%) and *Eimeria maxima* (3.2%). The prevalence and diversity of *Eimeria* spp. on farms producing backyard chickens in the semiarid region of Brazil were high, especially the diversity of species. Changing the management, with the adoption of sanitary measures, may be effective in reducing the high prevalence of coccidia on the farms studied.

## Introduction

Poultry production is one of the main livestock activities in the world and Brazil is one of the largest producers and consumers of chicken meat (1, 2). In tropical regions, production of backyard chickens is a family agriculture activity that is present on almost all farms and which forms part of the subsistence resources of small producers (3–5).

Poultry production is heavily affected by enteric diseases, which cause weight loss, increased mortality and low production rates and reduce the wellbeing of these animals (6). Among these enteric diseases, avian coccidiosis is the most important and prevalent worldwide (7, 8). Eight species of coccidia are known to affect chickens, namely: *Eimeria necatrix, Eimeria brunetti, Eimeria maxima, Eimeria praecox, Eimeria tenella, Eimeria mitis, Eimeria mivati* and *Eimeria acervulina* (9).

Most species of the genus *Eimeria* have different sites of infection. *Eimeria necatrix* and *E. tenella* are considered to be the most pathogenic species in chickens and infect the small intestine and cecum, respectively (10, 11). Mixed infections by different species can result in more severe presentations of the disease, as most species can affect different parts of the intestine (9, 12).

Although avian coccidiosis is the main enteric disease in chickens, few studies have described its prevalence or the diversity of species that infect backyard chickens, especially in tropical areas, such as the semiarid region of northeastern Brazil, therefore, this study aimed to evaluate the prevalence and diversity of *Eimeria* species in northeastern Brazil.

## Materials and methods

### Study location

The state of Paraíba, Brazil, has a total area of 58,584.6 km^2^, of which 86.2% (48,788.9 km^2^) belong to the semiarid region, with average temperatures of 27°C throughout the year and average precipitation of ~500 mm per year. There are usually two seasons: a rainy season from February to June, and a long dry season from July to January or occasionally lasting for more than a year (13). This study was conducted in the Sertão mesoregion, from February to August 2021.

### Sample population

The sampling plan used was a cross-sectional study and the sampling was designed to determine the prevalence of positive farms (foci). Sampling was carried out in two stages: (1) random selection of a pre-established number of farms (primary units); and (2) within the primary units, a pre-established number of chickens (secondary units) were randomly sampled.

The initial selection of farms was carried out through simple random sampling, as recommended by Thrusfield (14):


$$\begin{array}{l} n\;=\frac{z^{2}\cdot P\left(1-P\right)}{d^{2}} \end{array}$$

In which: *n*, number of farms selected; *z*, 1.96 (95% confidence level); P, expected prevalence (50%, to maximize the sample); *d*, standard error of 5%.

The local population was adjusted using the formula:


$$\begin{array}{l} n_{ajus}=\frac{N^{*}n}{N+n} \end{array}$$

n_ajus_, final number of farms selected; *n*, number of farms selected; *N*, number of farms existing.

The second stage was to determine the number of animals per farm based on detection of the disease in the herd, as prescribed by Thrusfield (14):


$$\begin{array}{l} n_{ani}=\left\{1-\left(1-p^{\frac{1}{d}}\right)\right\}\cdot \left\{N-\frac{d}{2}\right\}+1 \end{array}$$

In which: *n*_*ani*_, sample size required; *N*, farm population size; *d*, number of animals affected in the population (50%, to maximize the sample); *p*, probability of finding at least one case in the sample (95%).

**Figure 1:**
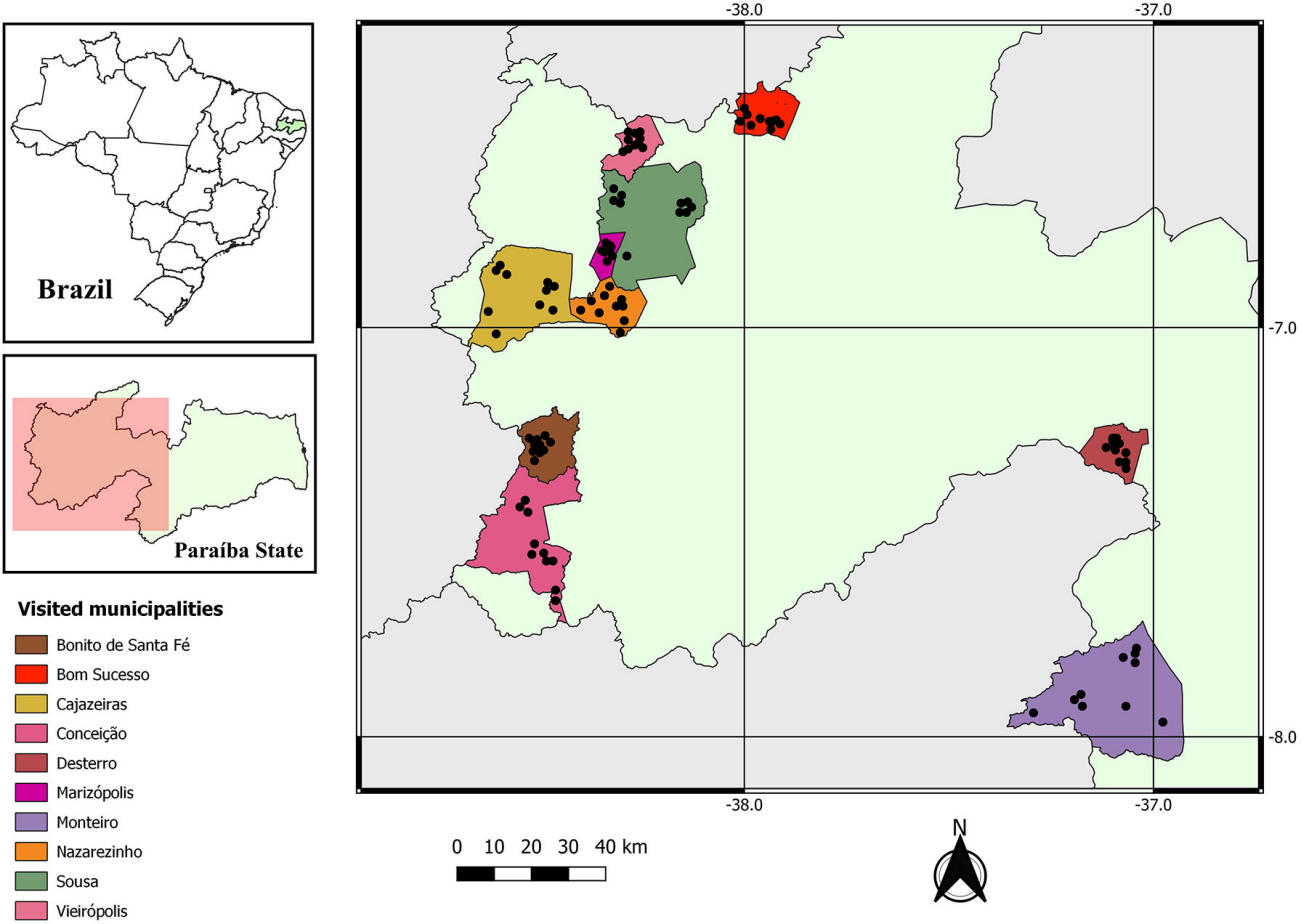
Geographical locations of the municipalities visited for diagnosing infection by *Eimeria* spp. in free-range chickens in the semiarid region of Paraíba, Brazil.

In the mesoregion studied, there are 99,545 chicken farms (15), among which 96 were needed (10% error) to make up the sample. However, samples were collected from 100 farms located in 10 municipalities (10 farms in each municipality): Sousa, Marizópolis, Nazarezinho, Monteiro, Cajazeiras, Bom Sucesso, Vieirópolis, Bonito de Santa Fé, Conceição and Desterro (Figure 1).

Regarding the animals, four samples were selected on farms that had up to 30 animals and five samples were selected on those that had more than 30 animals.

### Collection of samples for parasitological analysis

During the visits to the farms, fecal samples were collected directly from the rectal ampulla of poultry (*Gallus gallus*) in the growing phase, regardless of gender and breed. As the amount of feces collected from each animal rarely exceeded 2 g, which was insufficient to carry out parasitological analyses, samples from animals on the same farm were unified and homogenized, in order to form a single sample per farm. This material was sent to the Veterinary Parasitology Laboratory of the Instituto Federal de Educação, Ciência e Tecnologia da Paraíba (IFPB), Sousa Campus.

Samples from each farm were subjected to centrifuge-flotation examinations in sucrose solution, as described by Sheather (16) and adapted by Duszynski and Wilber (17). In the case of samples that were positive for coccidia, an aqueous solution of 2.5% potassium dichromate (K_2_Cr_2_O_7_) was added to the containers, such that the volumetric proportions were 16.7% feces and 83.3% potassium dichromate solution. These samples were then kept in a BOD incubator at an average temperature of 28°C for 15 days, for sporulation of the oocysts.

In the subsequent step, in order to remove excess potassium dichromate from the solution, the samples were placed in 50 ml centrifuge tubes and centrifuged at least four times for 10 min at a gravitational force of 1,050 × g, until they were completely clear. The sediment was then suspended by means of the centrifuge-flotation technique with a saturated sugar solution, at density 1.2, for 10 min at 1,050 × g. After centrifuging, supernatant drops were placed on a previously degreased and dried slide.

For the morphometric analysis, a LAB-DM300 digital microscope was used, coupled to a computer that was equipped with photomicrograph software suitable for the microscope used, which was capable of obtaining images with up to 3.2 million pixels. All photomicrographs were produced using 40 × and 100 × lenses (400 × and 1,000 × magnifications) and the measurements were made using the Mv Image^®^ software tools, similar to what was described by Araújo et al. (18) and Melo et al. (19).

The morphometric analysis was performed on sporulated and intact oocysts of the genus *Eimeria*. The polar diameter, equatorial diameter and shape index of the oocysts were obtained, in accordance with the values of Conway and Mackenzie (9). The reference values are shown in the Table 1. From each farm with at least one positive animal, 20 oocysts were evaluated, photographed and measured.

**Table 1:** Morphometric values of oocysts from free-range chickens that were obtained in the Sertão region of the state of Paraiba, northeastern Brazil.

**Species**	**SI**	**Polar diameter (μm)**	**Equatorial diameter (μm)**	**Oocyst shape**	**No. identified**	**Prevalence (%)**
*Eimeria necatrix*	1.23	17.9 ± 2.6^a^ (13.8–21.2)	14.6 ± 2.3^b^ (10.5–18.7)	Oblong ovoid	295	25
RV	1.19	13.2–22.7 μm	11.3–18.3 μm	Oblong ovoid		
*Eimeria mitis*	1.08	16.2 ± 2.4^a^ (10.3–19.1)	15.0 ± 3.0^c^ (9.8–18.4)	Subspherical	216	18.3
RV	1.09	11.7–18.7 μm	11.0–18.0 μm	Ellipsoid		
*Eimeria mivati*	1.16	16.1 ± 3.3^c^ (12.7–21.0)	13.9 ± 2.8^c^ (10.0–18.5)	Subspherical	204	17.3
RV	1.16	11.1–19.9 μm	10.6–16.2 μm	Ellipsoid		
*Eimeria tenella*	1.13	20.6 ± 3.5^b^ (17.4–24.7)	18.2 ± 3.1^b^ (15.7–21.3)	Ovoid	147	12.4
RV	1.16	19.5–26.0 μm	16.5–22.8 μm	Ovoid		
*Eimeria brunetti*	1.28	28.1 ± 5.1^b^ (20.4–33.6)	21.9 ± 3.9^b^ (15.4–26.3)	Ovoid	117	9.9
RV	1.31	20.7–30.3 μm	18.1–24.2 μm	Ovoid		
*Eimeria acervulina*	1.24	19.3 ± 3.6^b^ (15.4–23.7)	15.5 ± 3.0^b^ (12.1–18.9)	Ovoid	107	9.1
RV	1.25	17.7–20.2 μm	13.7–16.3 μm	Ovoid		
*Eimeria praecox*	1.28	21.2 ± 4.3^c^ (17.8–25.0)	16.6 ± 3.7^c^ (13.9–20.0)	Ovoid	56	4.8
RV	1.24	19.8–24.7 μm	15.7–19.8 μm	Ovoid		
*Eimeria maxima*	1.49	31.0 ± 5.8^b^ (23.0–36.7)	20.8 ± 3.7^b^ (15.1–23.1)	Ovoid	38	3.2
RV	1.47	21.5–42.5 μm	16.5–29.8 μm	Ovoid		
				Total	1180	100

SI, shape index; RV, Reference values [the reference values were obtained from Conway and McKenzie (9)].

Polar diameter and Equatorial diameter are shown in mean (minimum and maximum) values. Percentage of coefficient of variation (%CV) stated as “a” (%CV ≤ 15%); “b” (%CV > 15% < 20%) or “c” (%CV ≥ 20% < 25%).

### Data collection

On the farms visited, structured epidemiological questionnaires were applied to collect information about variables that may have an impact on infections. The variables investigated were the following: age group, breeding system, type of farm, poultry management, farm area, hygiene of feeders and drinkers, number of animals, clinical signs observed, use of anti-coccidial drugs, use of anti-coccidial vaccines, mortality rate and disease occurrence and prevention.

### Statistical analysis

The mean diameter, lower limit, upper limit, standard deviation and coefficient of variation (CV) of the oocysts of *Eimeria* spp. were evaluated using the Microsoft Office Excel 2010^®^ software. The analysis of associated factors for the infection with *Eimeria* spp. was conducted in two stages: univariate analysis and multivariable analysis. In the univariate analysis, each independent variable was crossed with a depedent variable, and those that presented *p* ≤ 0.20 using the chi-square test (χ^2^) or Fisher's exact test were selected for multivariate analysis using multiple logistic regression. The level of significance adopted in the multiple analyses was 5%. All analyses were performed with the SPSS software for Windows, version 20.0.

## Results

The prevalence of infections by *Eimeria* spp. on farms producing backyard chickens was 59% (59/100), with positive farms in all municipalities visited. On two farms, there was infection by one species of *Eimeria* spp.; on two farms, infection by two species; on 24 farms, infection by three different species; on 20 farms, infection by four species; and on 11 farms, infection by five species.

According to the morphological characteristics (their equatorial diameters, polar diameters and shape indexes) of the 1,180 sporulated oocysts examined, eight species of *Eimeria* spp. were diagnosed in the present study. Their morphological characteristics and respective prevalence are described in Table 1.

**Figure 2:**
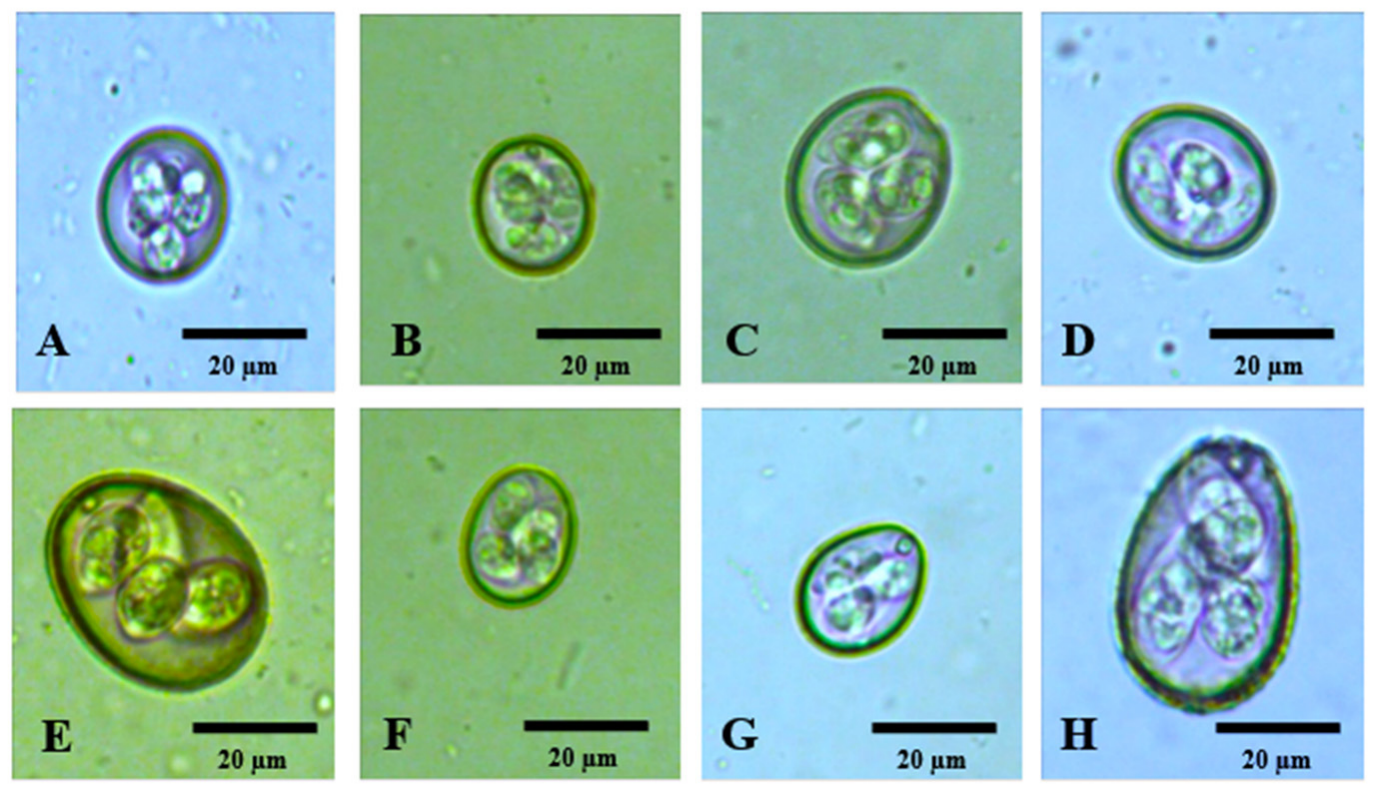
Photomicrographs of sporulated oocysts of *Eimeria* spp. of free-range chickens in the state of Paraíba, northeastern Brazil. **(A)** *Eimeria necatrix*; **(B)** *Eimeria mitis*; **(C)** *Eimeria mivati*; **(D)** *Eimeria tenella*; **(E)** *Eimeria brunetti*; **(F)** *Eimeria acervulina*; **(G)** *Eimeria praecox*; **(H)** *Eimeria maxima*. Magnification of 400×.

In decreasing order of prevalence, the species diagnosed were the following: *E. necatrix* (20), Figure 2A; *E. mitis* (21), Figure 2B; *E. mivati* (22), Figure 2C; *E. tenella* (23), Figure 2D; *E. brunetti* (24), Figure 2E; *E. acervulina* (21), Figure 2F; *E. praecox* (20), Figure 2G; and *E. maxima* (21), Figure 2H.

Production of free-range chickens was a way for the small-scale farmers to supplement their income on 42% (42/100) of the farms. Rudimentary installations were observed on almost all the farms. The feeding place for the chickens consisted of ordinary bowls on 65 farms, while the feed for the chickens was strewn on the ground on 33 farms and it was placed in appropriate feeders on only two farms. The water supplied to drinking fountains consisted of treated water on only four farms, while it was cistern water on 58 farms and it came from a weir on 38 farms. Anti-coccidial treatments were performed on 20% of the farms, made at least 3 months before the visits. Vaccinations against coccidiosis has not been done on the visited farms. None of the above variables was statistically significant (*p* ≤ 0.20) in the univariate analyses, therefore, no variables were selected for the multivariate analysis.

## Discussion

High prevalence of farms positive for *Eimeria* spp. was observed, 59, together with a diversity of eight species of *Eimeria*. in South Africa, Fatoba et al. (25) observed that 46.3% of the farms were positive for coccidian infections. In a similar study carried out in Tunisia by Kaboudi et al. (6), a prevalence of 31.8% was observed for coccidia of the genus *Eimeria* spp., and the species *E. maxima, E. tenella* and *E. acervulina* were characterized through their morphology. In Iran, Shirzad et al. (26) obtained lower diversity than that found in the present study, of five species of *Eimeria* spp. (*E. tenella, E. maxima, E. acervulina, E. brunetti* and *E. necatrix*) in broiler chickens.

In epidemiological surveys, oocyst morphology is still the most appropriate and reliable method for differentiation between *Eimeria* species (18, 27). This study was able to combine for the first time not only morphometric but also epidemiological data about free-range chicken infection by eight *Eimeria* species.

In addition to the fact that the animals in this study were being reared extensively, there are several other factors that may have influenced the prevalence and diversity of the oocysts found in this work. Nonetheless, management failures were probably the cause of the great diversity and prevalence, since most of these farms used water from ponds or wells, fed their animals on the ground or did not clean the water and/or food containers as often as needed. Some of the farms even had water containers close to or right below the perches on which the animals slept, thus leading to contamination of the water with feces.

In Japan, diversity more similar to that found in the present study was obtained by Matsubayashi et al. (28), who observed seven species of *Eimeria* spp (*E. acervulina, E. brunetti, E. maxima, E. mitis, E. necatrix, E. praecox* and *E. tenella*). The same authors also indicated that they had had difficulties in differentiating between the species, such that they observed six species of *Eimeria* spp. through the flotation technique and seven species by means of PCR. They noted that the species *E. maxima* and *E. necatrix* were difficult to differentiate visually. However, this was not seen in the present study, since *E. necatrix* had a characteristic oblong ovoid shape and much less shape index (Table 1) value than *E. maxima*, which made it easy to identify (Figure 2A) and *E. maxima* was the biggest ovoid oocyst present in the chickens (Figure 2H).

It was observed that the two largest species, *E. brunetti* and *E. maxima*, are easily distinguishable from all the others and the same happened to the two smallest species, *E. mitis* and *E. mivati*, are also very different from the others. It was also noted that *Eimeria* oocysts present homogeneous polar and equatorial diameter values, with low coefficient of variation (Table 1), meaning all the oocysts from each species presented similar sizes and shapes.

All the municipalities visited had farms with animals infected by *Eimeria* spp. This, together with the high diversity and the presence of species with high pathogenicity, demonstrates that there is a critical situation in this region. The high prevalences of *E. necatrix* (25%) and *E. tenella* (12.4%) indicate regular occurrence of coccidiosis caused by more pathogenic species, since their schizogony phases were seen to occur in the lamina propria of the intestinal crypts, thus causing great damage and intense hemorrhage (10).

There were no reports on the use of coccidiosis vaccination on visited farms. Nonetheless, promotion of parasite control through vaccinations is needed in this region, especially for the most pathogenic species, since use of vaccinations promotes restoration of parasite sensitivity to medications and promotes good immunity if used at the correct dosage (29, 30). Vaccinations can, however, make animals test positive, and oocysts are not visually different from environmental pathogenic oocysts, except for oocysts belonging to the species *E. brunetti* and *E. praecox*, for which no commercial vaccines exist (28).

The presence of mixed infections was observed on 57 farms, with infections by two to five species per farm. According to Flores et al. (31), the high prevalence and diversity, associated with indiscriminate use of anticoccidials are responsible for severe drug resistant *Eimeria* species in Korean chicken farms. Mixed infection, according to Fatoba et al. (25), is very common: it causes increased pathogenicity and is a threat to vaccine control. This information also demonstrates the failure of zootechnical control and the low quality of animal management, in which the animals lost weight. However, because they did not have diarrhea, their owners did not seek medical help or a way to deal with the problem.

There was high prevalence of coccidia of the genus *Eimeria* affecting chickens in the semiarid region of Paraíba, Brazil, and high variety of *Eimeria* species, with high prevalence rates for the most pathogenic species and for mixed infections on most positive farms, which may be favoring maintenance and spread of infections. Although not investigated in the present research, it is suspected that drug resistant *Eimeria* species already occurs. The study and development of production and management systems, to gain more knowledge about the sustainable control of coccidia in free-range chickens are necessary.

## Data availability statement

The raw data supporting the conclusions of this article will be made available by the authors, without undue reservation.

## Ethics statement

The animal study was reviewed and approved by Comitê de Ética no Uso de Animais - Instituto Federal de Educação, Ciência e Tecnologia da Paraíba. Written informed consent was obtained from the owners for the participation of their animals in this study.

## Author contributions

JS, FA, TF, and VV contributed to conception, design of the study, and wrote sections of the manuscript. EL, AS, GS, and BL organized the database. FA performed the statistical analysis. JS wrote the first draft of the manuscript. All authors contributed to manuscript revision, read, and approved the submitted version.

## Conflict of interest

The authors declare that the research was conducted in the absence of any commercial or financial relationships that could be construed as a potential conflict of interest.

## Publisher's note

All claims expressed in this article are solely those of the authors and do not necessarily represent those of their affiliated organizations, or those of the publisher, the editors and the reviewers. Any product that may be evaluated in this article, or claim that may be made by its manufacturer, is not guaranteed or endorsed by the publisher.
